# Polymicrobial biofilms of ocular bacteria and fungi on ex vivo human corneas

**DOI:** 10.1038/s41598-022-15809-z

**Published:** 2022-07-08

**Authors:** Konduri Ranjith, Banka Nagapriya, Sisinthy Shivaji

**Affiliations:** Jhaveri Microbiology Centre, Prof. Brien Holden Eye Research Centre, L. V. Prasad Eye Institute, Kallam Anji Reddy Campus, Hyderabad, Telangana 500034 India

**Keywords:** Antimicrobials, Biofilms

## Abstract

Microbes residing in biofilms confer several fold higher antimicrobial resistances than their planktonic counterparts. Compared to monomicrobial biofilms, polymicrobial biofilms involving multiple bacteria, multiple fungi or both are more dominant in nature. Paradoxically, polymicrobial biofilms are less studied. In this study, ocular isolates of *Staphylococcus aureu*s, *S. epidermidis* and *Candida albicans*, the etiological agents of several ocular infections, were used to demonstrate their potential to form mono- and polymicrobial biofilms both in vitro and on human cadaveric corneas. Quantitative (crystal violet and XTT methods) and qualitative (confocal and scanning electron microscopy) methods demonstrated that they form polymicrobial biofilms. The extent of biofilm formation was dependent on whether bacteria and fungi were incubated simultaneously or added to a preformed biofilm. Additionally, the polymicrobial biofilms exhibited increased resistance to different antimicrobials compared to planktonic cells. When the MBECs of different antibacterial and antifungal agents were monitored it was observed that the MBECs in the polymicrobial biofilms was either identical or decreased compared to the monomicrobial biofilms. The results are relevant in planning treatment strategies for the eye. This study demonstrates that ocular bacteria and fungi form polymicrobial biofilms and exhibit increase in antimicrobial resistance compared to the planktonic cells.

## Introduction

The surface of the eye harbors a community of bacteria, fungi, and viruses which under normal conditions are harmless. Several of these microorganisms have been identified as the etiological agents of ocular diseases such as conjunctivitis, keratitis, endophthalmitis, blepharitis, orbital cellulitis, dacryocystitis etc.^1^. These ocular infections are susceptible to antibacterials, antifungals and antivirals. But treating ocular diseases is complicated by factors such as: infections of the eye that could be either monomicrobial or polymicrobial^2^ and additionally the occurrence of antimicrobial resistance (AMR) in the microbes. A strategy commonly used by microbes to become resistant to antimicrobials is the ability of the microbes to form a biofilm^3,4^ which protects the microbes from the hostile environmental conditions and simultaneously the microbes exhibit metabolic cooperation, acquire AMR phenotypes, show altered expression of virulence genes and virulence factors^5–9^. Biofilm formation is extremely relevant to human health and is associated with 80% of human infections as in cystic fibrosis^10^, otitis^11^, sinusitis^12^, diabetes wound infection^13^ etc. Biofilm formation is also observed on indwelling medical devices (IMDs) such as intravenous catheters^14^, prosthetic heart valves^15^, orthopaedic devices^16^, contact lenses^17^, etc. Further, a biofilm could also be polymicrobial involving cohabitation of a bacterium and a fungus, or two different bacteria, or two different fungi^3^ or more^18^. Polymicrobial biofilms are more challenging to treat since they are more resistant to antimicrobial treatment than the corresponding single-species biofilms^19,20^ and corresponding planktonic cells^21^.

Studies have indicated that the ocular bacterial pathogens *Pseudomonas aeruginosa*, *Staphylococcus aureus, S. epidermidis*, *Streptococcus* spp., *Enterobacter* spp., *E. agglomerans*, *Micrococcus luteus, Serratia marcescens, Neisseria* spp*., Moraxella* spp*., Bacillus* spp*., Escherichia coli, Proteus mirabilis*, and *Klebsiella* spp.^22,23^, exhibit the potential to form biofilms on intraocular lenses, contact lenses, suture material, lid implants, socket implants, orbit implants and scleral buckles^24^. Further, the antibiotic required for killing the cells in the biofilm phase is greater than 100 fold than that required for killing planktonic cells^25^. These in vitro studies on monomicrobial biofilms need to be compared with polymicrobial biofilms involving multiple bacteria, bacteria and fungi and maybe algae and protozoa^26^. Polymicrobial biofilms was first described for bacteria residing in the oral cavity^27,28^ and chronic wounds^29^ and indicated that direct extrapolations from monomicrobial biofilms in vitro to polymicrobial biofilms are imprecise and misleading with respect to the protective effect of the biofilms, virulence enhancement and horizontal gene transfer in the biofilm^30^. Polymicrobial biofilms are still poorly described.

This study reports that ocular *S. aureus* and *S. epidermidis* isolated from two vitreous samples from patients with endophthalmitis^31^, and the fungus *Candida albicans* obtained from patients with microbial keratitis^32^, could form mixed polymicrobial biofilms in which resistance to antimicrobial agents is increased several fold compared to monomicrobial biofilms and planktonic cells. The study combines in vitro results using tissues culture plates and ex vivo results using human cadaveric cornea as the substratum for biofilm formation. This study reports that ocular *S. aureus* and *S. epidermidis* isolated from two vitreous samples from patients with endophthalmitis^31^ and the fungus *Candida albicans* obtained from patients with microbial keratitis^7,32^ could form polymicrobial mixed biofilms in which resistance to antimicrobial drugs is increased several folds compared to the planktonic cells.

## Materials and methods

### Study centre

The L V Prasad Eye Institute (LVPEI) is a comprehensive eye health facility in India and is recognized as a World Health Organization Collaborating Centre for Prevention of Blindness.

### Cultivation of the bacterial isolates

Vitreous fluid samples of two patients with endophthalmitis when cultured on 5% sheep blood agar medium plates^33^ yielded two single colonies. These two colonies were purified by repeated streaking and characterised by biochemical methods and 16S rRNA gene sequencing as described earlier^6,31^. Isolate L-1058-2019(2) produced pink color colonies on MSA agar, white opaque color colonies on non-hemolytic blood agar and was negative for coagulase and oxidation-fermentation test, suggestive of *Staphylococcus epidermidis*. In contrast, isolate L-1054-2019(2) was yellow pigmented on MSA agar and positive for coagulase and oxidation-fermentation test suggestive of *Staphylococcus aureus*. The identity of the two isolates was also confirmed using Vitek 2 Compact System (BioMérieux, Marcy l’Etoile, France). The two isolates were preserved in tryptone soya broth [TSB containing Tryptone (17%), Soy (3%), NaCl (2.5%), K_2_HPO_4_ (2.5%), glucose (2.5%)]^33^ with 30% glycerol at − 80 °C and routinely cultured on 5% sheep blood agar plates by overnight incubation at 37 °C^6,31^.

### Cultivation of the fungal isolate

The fungus was isolated from the corneal scrapings of a patient with keratitis and identified as *Candida albicans* (L-391-2015) using a Vitek 2 compact system employing YST strips (BioMérieux, Marcy l’Etoile, France) and by ITS1 and ITS2 gene sequencing as described earlier^32^. *C. albicans* was preserved in TSB^33^ as above and was routinely grown in Sabouraud dextrose medium (SDM) (dextrose (20%) and peptone (10%) and final pH adjusted to 5.6) at 30 °C.

### Biofilm formation in ocular bacteria and fungi by the tissue culture plate method using crystal violet method

Biofilm formation was monitored in ocular isolates of *S. aureus* (L-1054-2019(2)), *S. epidermidis* (L-1058-2019(2)) and *C. albicans* (L-391-2015) by the tissue culture plate method (TCP) using crystal violet (CV) as described earlier^6,31^. In the CV method an overnight culture in YPD medium [(bacteriological peptone (20%), glucose(20%) and yeast extract (20%)] was diluted 10,000 times (v/v) and then 100 µl of the suspension (10^4^ cells/ml) was incubated in a 96 well plate containing 100 µl of YPD medium at 37 °C for 24 h and 48 h. After incubation, the YPD medium was decanted, the planktonic cells discarded, and the cells that adhered to the wells were washed twice with 200 µl of phosphate-buffered saline (PBS), pH 7.4 (1× PBS contains 137 mM NaCl, 2.7 mM KCl, 10 mM Na_2_HPO_4_ and 1.8 mM KH_2_PO_4_), and plates air dried at room temperature. The bacterial cells that had adhered to the wells were stained using 0.1% aqueous crystal violet (Sigma Chemical Co., St. Louis, MO, USA). Excess crystal violet was discarded, and each well was washed twice with 200 µl of PBS and dried at RT. CV associated with the bacteria was extracted with 200 µl of absolute ethanol and quantified using a Spectrophotometer [SpectraMax M3, with a cuvette adaptor (Molecular Devices, San Jose, CA, USA)] set at 595 nm. Wells without cells served as the control (OD was < 0.1 at 595 nm) and the OD value was deducted from the “high-biofilm formers” (OD > 0.3 at 595 nm) and “low-biofilm formers” (OD < 0.3 at 595 nm)^32,33^. *S. aureus* ATCC25922 (positive for biofilm formation) and *E. coli* ATCC25923 (negative for biofilm formation) served as a positive and negative controls respectively for biofilm formation. The experiment was performed with three replicates.

### Biofilm formation in ocular bacteria and fungi by the tissue culture plate method using XTT

In the XTT [2,3-Bis-(2-methoxy-4-nitro-5-sulfophenyl)-2H-tetrazolium-5-carboxanilide] (Sigma, USA) method^7,37^, cultures in YPD medium were diluted 10,000 times with YPD and then 100 µl of this suspension (10^4^ cells/ml) was incubated in YPD in a 96 well plate for 24 h and 48 h. The media was then decanted, each well washed twice using 200 µl of autoclaved milliQ water and allowed to air dry for 30 min.The washed cells were stained in the dark with XTT by adding 200 µl of XTT solution [147 µl of PBS and 50.5 µl of XTT (1 mg/ml, Sigma Chemical Co., St. Louis, MO, USA) and 2.5 µl of Menadione (0.4 mM, Sigma Chemical Co., St. Louis, MO, USA)] and incubated in the dark at 37 °C for 3 h. From each well, 100 µl was then transferred to a fresh 96 well plate and biofilm formation was quantified using a SpectraMax M3, microplate reader (Molecular Devices, CA, USA)^7,37^. Media without cells served as a negative control (OD was < 0.1 at 595 nm) and *S. aureus* ATCC 25922 (positive for biofilm formation) and *E. coli* ATCC 25923 (negative for biofilm formation) served as a negative and positive control respectively for biofilm formation with OD values of < 0.3 and > 0.3 respectively were considered as low-biofilm formers and high-biofilm formers.

### Polymicrobial biofilm by simultaneous incubation of bacteria and fungi

In polymicrobial biofilm formation more than one microorganism is monitored for biofilm formation. *C. albicans* along with either *S. aureus* or *S. epidermidis* were co-incubated in YPD media at 37 °C for 24 and 48 h at a final volume of 100 µl containing 10^4^ cells/ml of the fungus and the bacterium. After incubation, the wells were stained with CV or XTT and biofilm quantified as described above.

### Polymicrobial biofilm formation when bacteria and fungi were not incubated simultaneously

The bacterium or the fungus (10^4^ cells/ml) was allowed to form a biofilm for 24 h after which planktonic cells of bacteria or fungi was added to the preformed biofilm which was allowed to grow for another 24 h. After incubation, the wells were stained with CV or XTT and biofilm quantified as described above.

### Biofilm thickness in monomicrobial and polymicrobial biofilms on human cadaveric corneas using confocal laser scanning microscopy

Monomicrobial and polymicrobial biofilms of ocular bacteria and the fungus were set up on on human cadaveric corneas as in the CV and XTT methods^34^ and used for localisation of extracellular polymeric substance (EPS) and for the determination of the thickness of the biofilm by Confocal Laser Scanning Microscopy (CLSM) using dual staining^8^. After the incubation period of 24 and 48 h in RPMI medium, the human cadaveric corneas were washed with autoclaved distilled water and fixed with 250 µl of formaldehyde (4%) for 3 h. Fixed biofilms were then washed twice with autoclaved distilled water as above and stained for 30 min with 200 µl of 1.67 µM Syto9 (Invitrogen, Carlsbad, CA, USA), a nuclear fluorescent dye that stains DNA of viable cells and emits green color. After staining with Syto 9, biofilms were stained in the dark with 0.025% Calcofluor white M2R (Sigma Chemical Co., St. Louis, MO, USA) for 30 min. This dye binds to β-linked polysaccharides and fluoresces under long-wave UV light and biofilm could be visualized (blue) using confocal microscopy (Carl Zeiss LSM 880, Jena, Germany)^37^. The Argon Laser was excited at 450–490 nm for Syto9 and 363-nm using a 455/30 band-pass filter for Calcofluor white and a 20× objective was used set at Zoom 2. The thickness of the biofilm at each time point was measured across the biofilm and values are reported as Z axis, average ± standard deviation in µm. The data was analysed statistically using unpaired t-test. p value of < 0.05 was considered significantly different.

### Visualisation of monomicrobial and polymicrobial biofilms on human cadaveric cornea using scanning electron microscopy

The procedure is identical to that described earlier^37^. Human cadaveric cornea which did not meet the stringent quality required for transplantation were obtained from The Ramayamma International Eye Bank (RIEB), LVPEI, Hyderabad, India. All corneas were obtained following procedures approved by the institutional review board for the protection of human subjects. Corneas were received in MK medium containing gentamicin^31^. These corneas were thoroughly washed with PBS prior to use for biofilm formation. The donor cornea with its epithelial surface facing upward was immersed in an antibiotic free RPMI (Roswell Park Memorial Institute) media^31,35^ containing 10% fetal calf serum, 5 μg/ml insulin and 10 ng/ml epidermal growth factor and incubated for 24 h at 37 °C in a 5% CO_2_ incubator to remove the residual antibiotics. Corneas from the antibiotic free RPMI medium were washed with PBS and a sterile steel scalpel was used to create three horizontal and vertical cuts^31,35^. Subsequently, the bacterial and fungal inoculum prepared from an overnight culture grown in YPD broth was diluted 10,000 times with YPD broth and centrifuged at 12,000 rpm (Eppendorf USA, Framingham, MA, USA, model no: 5430) for 5 min at room temperature (25 °C) and the pellet washed with 200 μl of autoclaved distilled water and centrifuged. The final pellet was suspended in 100 μl of RPMI and was gently transferred onto the surface of the corneas and incubated for 24 or 48 h at 37 °C in a CO_2_ incubator (5% CO_2_ in air). After the incubation period, the corneas were processed for SEM to visualize biofilms on the cornea. For this purpose,the corneas were washed with PBS, fixed with 2.5% glutaraldehyde (Himedia-Secunderabad, India) and washed again prior to dehydration through graded ethanol (10, 25, 50, 70, 90 and 100% for 20 min each) and finally air dried overnight. Biofilms on the corneas were sputtered with gold for 60 s using a high vacuum evaporator (SC7620 PALARON Sputter Coater, East Sussex, UK) and visualized using a scanning electron microcope (SEM) (Carl Zeiss-Model EVO 18, Carl Zeiss, Germany). The voltage used for acquiring the SEM images ranged between 5 and 20 kV^31^.

### Antimicrobial susceptibility in planktonic phase

Several antimicrobials (antifungals and antibacterials) were evaluated for their antimicrobial activity as per Clinical and Laboratory Standards Institute (CLSI) (drivingitproductivity.com/2021/10/28/clsi-guidelines-for-antimicrobial-susceptibility-testing/). An overnight bacterial suspension in YPD broth was adjusted to 0.5 McFarland units, diluted 100-fold and 100 µl of the suspension was added to each well of a 96 well polystyrene plate (Nunclon™, Thermo Scientific, Roskilde, Denmark) containing 100 µl of an antifungal/antibacterial agent of a known concentration. Minimum inhibitory concentration (MIC/MBEC) for each antimicrobial agent was determined in three replicates according to CLSI-M07-A10 guidelines as described earlier^6–8,31^.

### Antibiotic susceptibility in monomicrobial biofilms

Inhibitory effects of antimicrobials on monomicrobial biofilms was performed as described earlier^6–8,31^. Briefly, an overnight culture of bacterium or fungus in YPD medium was diluted (10^4^ cells/ml) and was allowed to form a biofilm at 37 °C for 24 h as described above. The YPD medium was decanted, the wells washed twice with PBS to remove the planktonic cells and the required concentration of the antimicrobial agent was added. After incubation for additional 24 h the wells were washed with 200 µl of PBS to remove the planktonic cells and the plates were then processed for monitoring the effect of the antimicrobial agent by the XTT method^6–8,31^. Inoculums without the addition of the compound served as a negative control. All experiments were performed in triplicate.

### Antibiotic susceptibility in polymicrobial biofilms

Bacteria plus fungi were incubated simultaneously in YPD to form a biofilm for 24 h after which the planktonic cells were discarded, the biofilm washed twice with PBS and then the antibacterial or antifungal agent was added for an additional 24 h. Subsequently, the biofilms were washed, and the effect of the antimicrobial agent was evaluated by the XTT method^31^.

In a separate experiment either the bacterium or the fungi were allowed to form a biofilm for 24 h after which the other was added and additionally incubated for another 24 h. At the end of the 48 h incubation period the biofilms were washed and then the antibacterial or antifungal agent was added for an additional 24 h. Subsequently, the biofilms were washed, and the effect of the antimicrobial agent was evaluated by the XTT method as described^31^. Inoculums without the addition of the compound served as a negative control. All experiments were performed in triplicate.

### Statistical analysis

All experiments were performed in triplicates and standard deviation was calculated. Wherever applicable all comparisons were evaluated using unpaired t test for proportions and homogeneity and a p ≤ 0.05 was considered significant.

### Ethical approval

All experiments were performed in accordance with relevant guidelines and regulations of the L V Prasad Eye Institute, Hyderabad, India and the experimental protocols were approved by the Institutional Review board and the Institutional ethics committee of the L V Prasad Eye Institute, Hyderabad, India (LEC-BHR-P-04-21-623). Additionally, informed consent was obtained from the legal guardians of all the donors. All the donors were adults and in the age group of 22–35 years.

## Results

In this study both the XTT and CV methods were used for quantification of the biofilms. The XTT method measures cell viability^31^ whereas the CV method measures cell wall material and biofilm matrix^31^.

### Quantification of polymicrobial biofilms of bacteria and fungi by the XTT method

**Figure 1:**
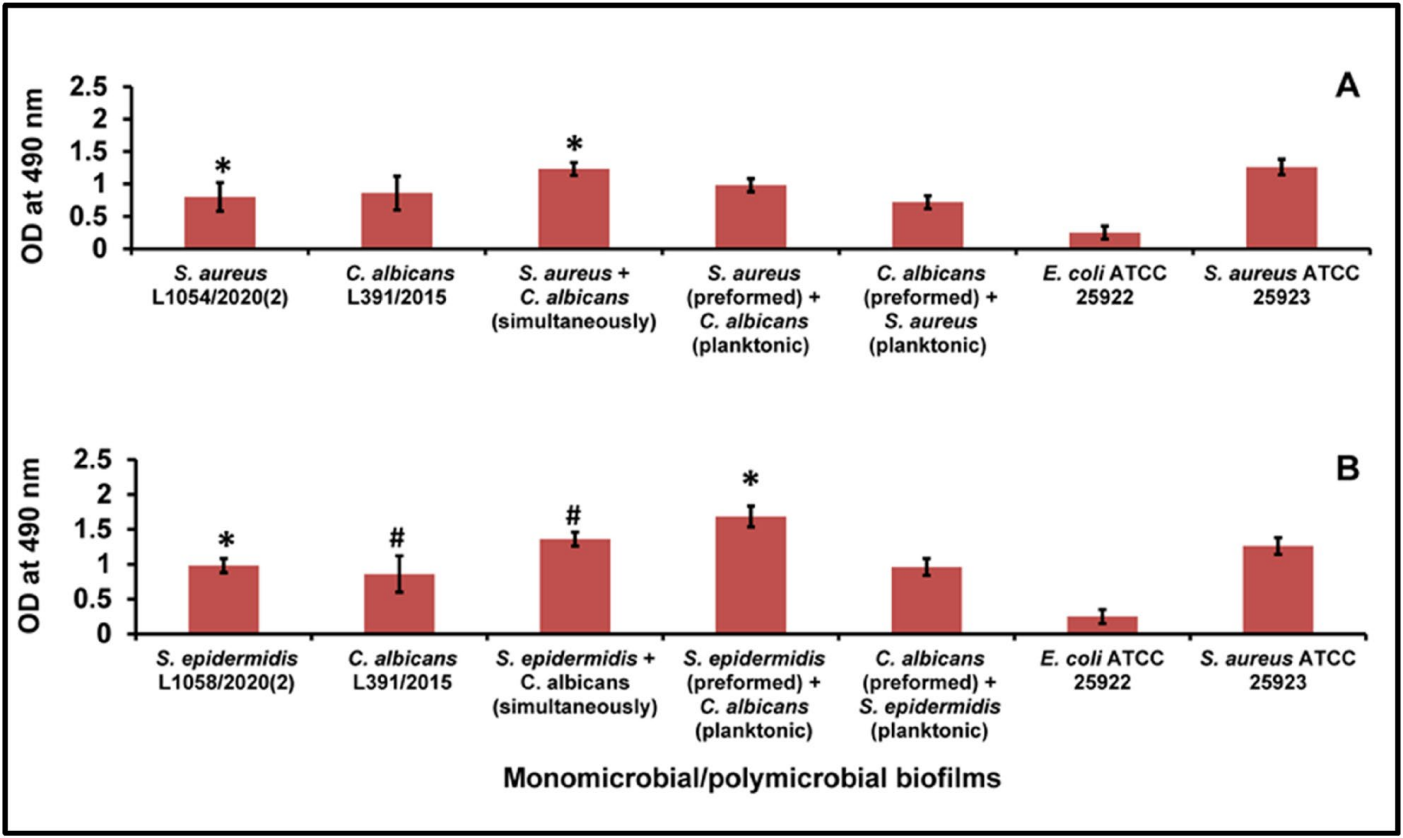
Quantification of polymicrobial biofilm formation in *S. aureus* (**A**) and *S. epidermidis* (**B**) with *C. albicans* by the XTT method after 48 h of biofilm formation compared to the monomicrobial biofilms of *S. aureus*, *S. epidermidis* and *C. albicans*. Similar superscripts * and ^#^ indicate significant increase (p value < 0.05) in polymicrobial biofilm compared to the respective monomicrobial biofilm at 48 h. Unpaired t test was used for the calculation of p value. *S. aureus* ATCC 25923 was used as a positive control and *E. coli* ATCC 25922 was used as a negative control. Experiments were performed in triplicates. Values represent XTT absorbance at 495 nm expressed as average ± standard deviation.

The quantification of the biofilms by the XTT method was done after 24 and 48 h of biofilm formation and consistently the biofilms of *S. aureus, S. epidermidis* and *C. albicans* exhibited an increase in XTT OD at 48 h compared to 24 h of biofilm formation (Supplementary table 1). For brevity, the results of changes observed after 48 h biofilm formation are presented (Fig. 1). In the polymicrobial biofilms involving *S. aureus* and *C. albicans* incubated simultaneously, significant increase in biofilm formation was observed at 48 h in the polymicrobial biofilm over the corresponding monobacterial biofilm of *S. aureus* (Fig. 1A; Supplementary table 1). The polymicrobial biofilm of *S. epidermidis* plus *C. albicans* when incubated simultaneously exhibited significant increase over the monofungal biofilm of *C. albicans* (Fig. 1B; Supplementary Table 1). Increase was more pronounced in *S. aureus* plus *C. albicans* polymicrobial biofilm at 48 h compared to 24 h biofilm formation when the OD doubled (Supplementary Table 1). Further, when *C. albicans* was added to preformed biofilms of *S. epidermidis* significant increase in XTT positive (metabolically active cells) was consistently observed compared to the monomicrobial biofilm of *S. epidermidis* at 48 h (Fig. 1B; Supplementary Table 1). Thus, polymicrobial biofilms are more pronounced compared to the monomicrobial biofilms with respect to metabolically active cells in the biofilm (also see supplementary Table 1).

### Quantification of polymicrobial biofilms of bacteria and fungi by the tissue culture plate method using crystal violet

**Figure 2:**
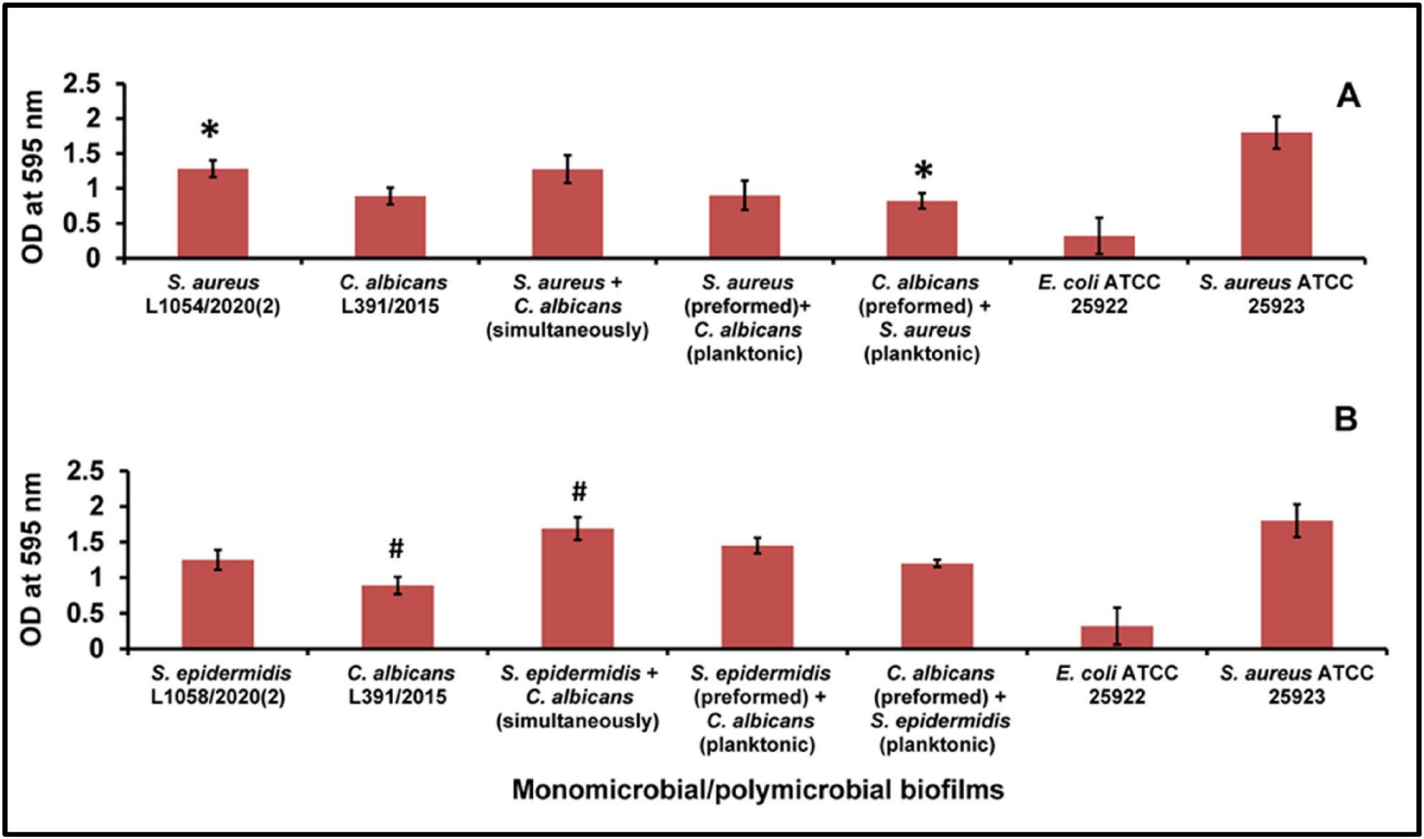
Quantification of polymicrobial biofilm formation in *S. aureus* (**A**) and *S. epidermidis* (**B**) with *C. albicans* by the CV method after 48 h of biofilm formation compared to the monomicrobial biofilms of *S. aureus*, *S. epidermidis* and *C. albicans*. Similar superscripts * and ^#^ indicate significant increase (p value < 0.05) in polymicrobial biofilm compared to the respective monomicrobial biofilm at 48 h. Unpaired t-test was used for the calculation of p value. *S. aureus* ATCC 25923 was used as a positive control and *E. coli* ATCC 25922 was used as a negative control. Experiments were performed in triplicates. Values represent crystal violet absorbance at 595 nm expressed as average ± standard deviation.

In the TCP method using crystal violet (CV) only the polymicrobial biofilm wherein *C. albicans* biofilm was preformed and then *S. aureus* was added, significant increase was observed only with the monomicrobial *S. aureus* biofilm (Fig. 2A; Supplementary Table 2). The polymicrobial biofilm of *S. epidermidis* and *C. albicans* incubated simultaneously also showed significant increase in the OD of CV compared to the monofungal biofilm (Fig. 2B; Supplementary Table 2).

### Biofilm thickness in monomicrobial and polymicrobial biofilms on human cadaveric corneas using confocal laser scanning microscope

**Figure 3:**
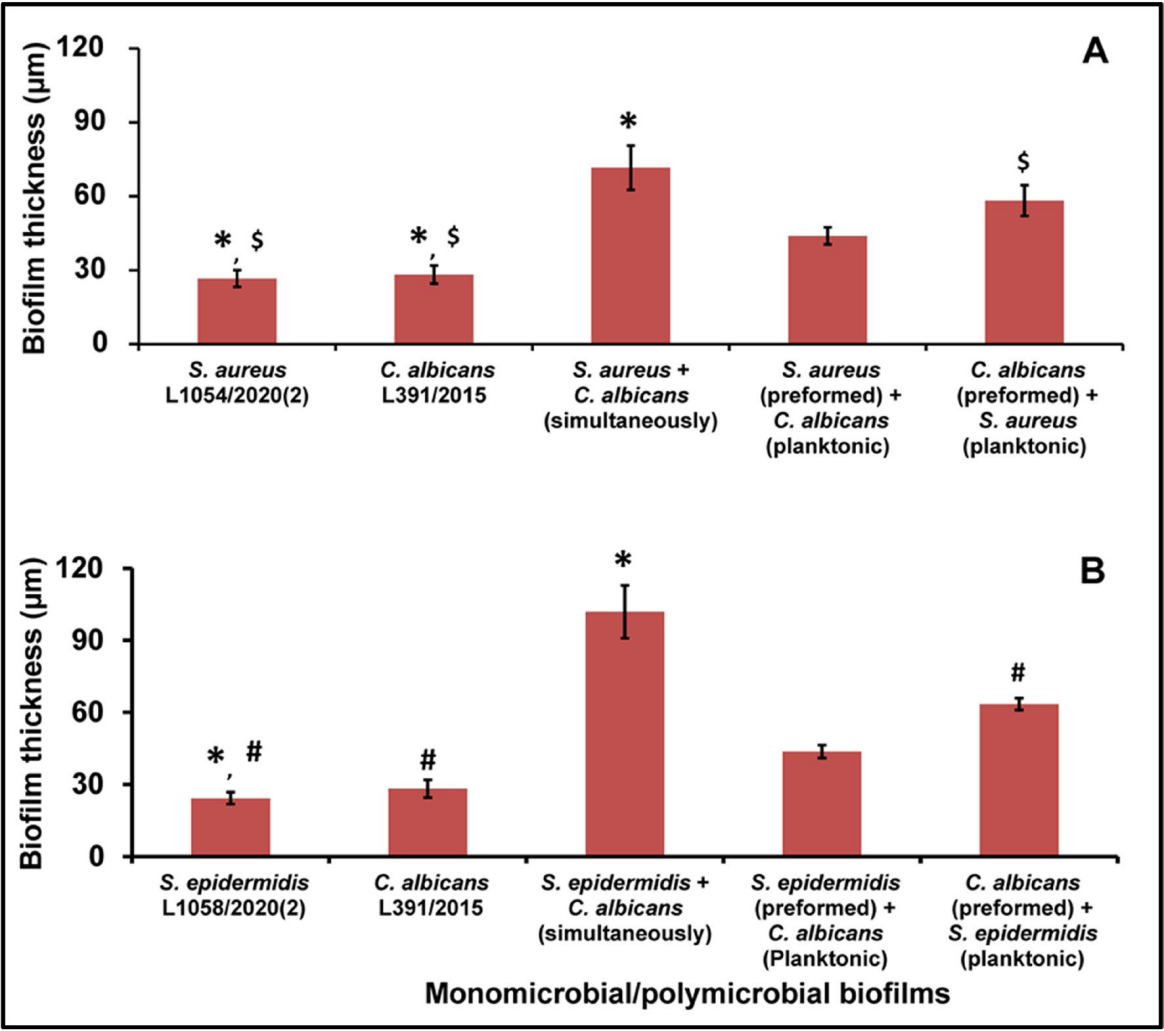
Measurement of polymicrobial biofilm thickness in ocular *S. aureus* (**A**) *S. epidermidis* (**B**) and *C. albicans* (**A**,**B**) after 48 h of biofilm formation by confocal laser scanning microscopy. Similar superscripts *, ^#^ and ^$^ indicate significant increase (p value < 0.05) in polymicrobial biofilm compared to the respective monomicrobial biofilm at 48 h. Unpaired t test was used for the calculation of p value. *S. aureus* ATCC 25923 was used as a positive control and *E. coli* ATCC 25922 was used as a negative control. Experiments were performed in triplicates.

The thickness of the monomicrobial biofilms of *S. aureus, S. epidermidis* and *C. albicans* increased significantly from 24 to 48 h (Supplementary Figs. 1, 2a,e; b,f). Polymicrobial mixed biofilms of *S. aureus* with *C. albicans* showed significant increase in thickness when they were simultaneously incubated (Fig. 3A, supplementary Figs. 1, 2c,g) compared to both monobacterial and monofungal biofilms at 48 h. In addition, when the polymicrobial biofilm was generated by preforming the *C. albicans* biofilm to which *S. aureus* was added also showed significant increase in thickness over both the monobacterial and monofungal biofilms (Fig. 3A, Supplementary Figs. 1, 2c,g).

*Staphylococcus epidemidis* and *C. albicans* polymicrobial biofilm incubated simultaneously showed significant increase in biofilm formation compared to monobacterial biofilm at 48 h. Polymicrobial biofilm of *C. albicans* to which *S. epidermidis* was added also showed increase in biofilm formation compared to both monobacterial and monofungal biofilms (Fig. 3B, Supplementary Figs. 1, 2).

### Visualisation of monomicrobial and polymicrobial biofilms of *S. aureus* and *C. albicans* on human cadaveric cornea using scanning electron microscopy

**Figure 4:**
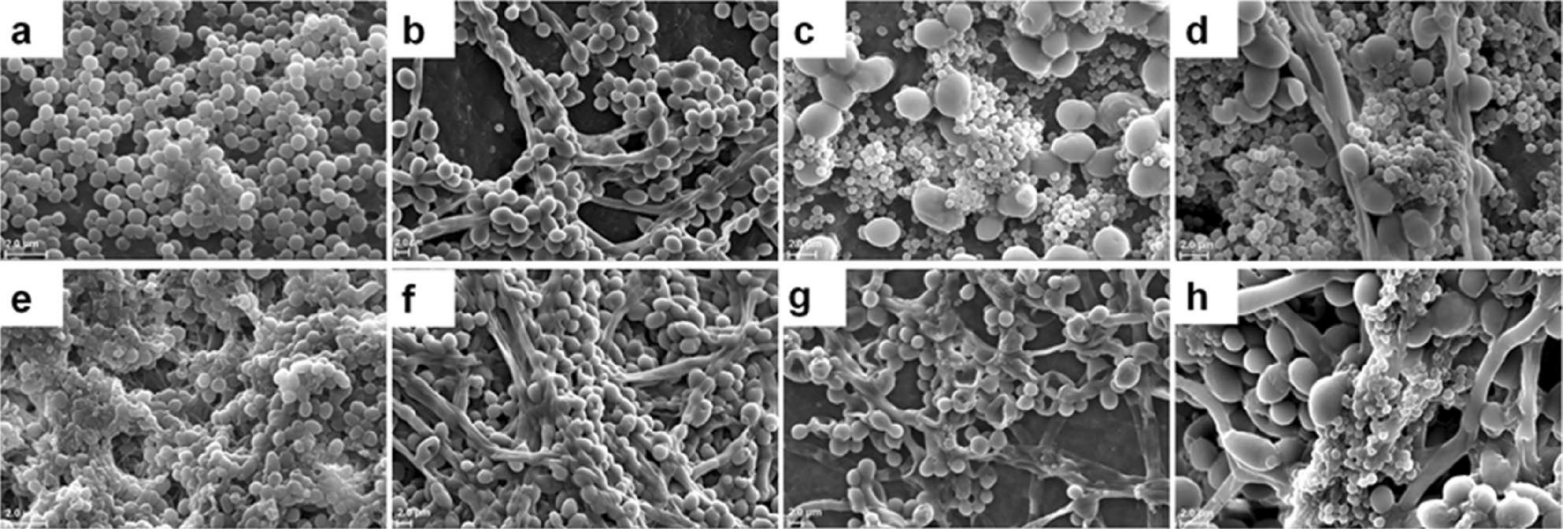
Visualisation of monomicrobial and polymicrobial biofilms of *S. aureus* and *C. albicans* on human cadaveric cornea using Scanning Electron Microscopy. *S. aureus* biofilm at 24 h (**a**), *C. albicans* biofilm at 24 h (**b**), polymicrobial mixed biofilm of *S. aureus* and *C. albicans* grown simultaneously for 24 h (**c**), preformed biofilm of *S. aureus* for 24 h to which *C. albicans* was added, (**d**) *S. aureus* biofilm at 48 h (**e**), *C. albicans* biofilm at 48 h (**e**), polymicrobial mixed biofilm of *S. aureus* and *C. albicans* grown simultaneously for 48 h (**g**) and preformed biofilm of *C. albicans* for 24 h to which *S. aureus* was added (**h**).

*Staphylococcus aureu*s and *C. albicans* formed monomicrobial biofilms by 24 h and multilayer clumping of cells was visible, and EPS was sparingly seen (Fig. 4a,b). In *C. albicans* at 24 h hyphae were also visible (Fig. 4b). When cultured together at 24 h both *S. aureus* and *C. albicans* formed polymicrobial mixed biofilms with *S. aureus* forming small clumps on the surface of *C. albicans* (Fig. 4c). Further when *C. albicans* was added to 24 h preformed biofilm of *S. aureus* polymicrobial mixed biofilms were formed and clumping of both the bacteria and fungi was more intense, hyphae were more prominent, and EPS was clearly visible (Fig. 4d) compared to when the bacterium and fungi were cultured together to form the polymicrobial biofilm (Fig. 4c).

By 48 h the monomicrobial biofilms of *S. aureus* and *C. albicans* formed denser clumps and produced more EPS compared to 24 h of monomicrobial biofilms (Fig. 4e,f). Further, when *S. aureus* and *C. albicans* were grown simultaneously for 48 h the polymicrobial biofilms exhibited clumping and the *S. aureus* were totally enclosed in EPS (Fig. 4g). But, when *C. albicans* monomicrobial biofilm was preformed for 24 h and then *S. aureus* was added the resulting polymicrobial mixed biofilms exhibited intense clumping of both the bacteria and fungi, hyphae were more prominent and EPS was clearly visible (Fig. 4h).

### Visulisation of monomicrobial and polymicrobial biofilms of *S. epidermidis* and *C. albicans* on human cadaveric cornea using scanning electron microscopy

**Figure 5:**
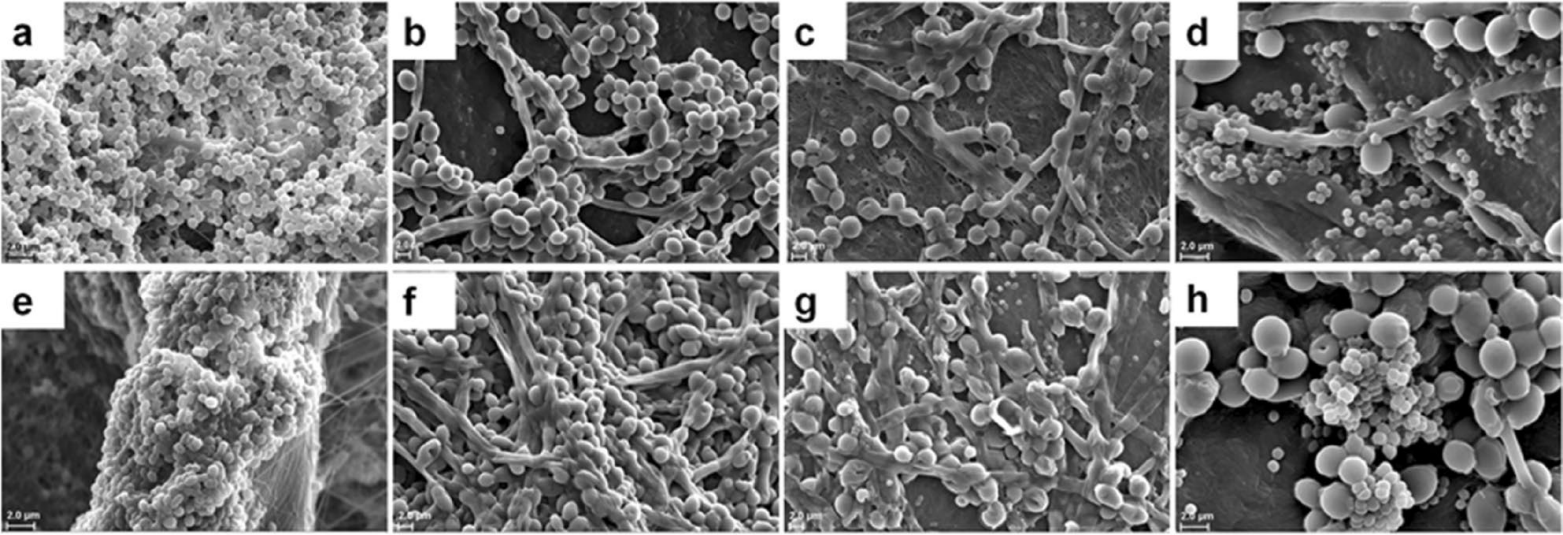
Visualisation of monomicrobial and polymicrobial biofilms of *S. epidermidis* and *C. albicans* on human cadaveric cornea using scanning electron microscopy. *S. epidermidis* biofilm at 24 h (**a**), *C. albicans biofilm* at 24 h (**b**), polymicrobial mixed biofilm of *S. epidermidis* and *C. albicans* grown simultaneously for 24 h (**c**), preformed biofilm of *S. epidermidis* for 24 h to which *C. albicans* was added (**d**), *S. epidermidis* biofilm at 48 h (**e**), *C. albicans* biofilm at 48 h (**f**), polymicrobial mixed biofilm of *S. epidermidis* and *C. albicans* grown simultaneously for 48 h (**g**) and preformed biofilm of *C. albicans* for 24 h to which *S. epidermidis* was added (**h**).

*Staphylococcus epidermidis* like *S. aureus* formed monomicrobial biofilms which appeared as multi-layered clumps of cells and EPS was visible (Fig. 5a). The biofilm by 48 h appeared as huge column of multilayered biofilm covered with EPS (Fig. 5e compared with Fig. 5a). But when *S. epidermidis* was simultaneously induced to form biofilm along with *C. albicans,* the biofilm was not very prominent at 24 h (Fig. 5c) but it was more dense and only a few bacteria were visible at 48 h (Fig. 5g). Further when *S. epidermidis* was cultured for 24 h and then *C. albicans* was added, clumping of the two microbes was observed separately and a few colonised the surface of *C. albicans* hyphae and yeast forms (Fig. 5d). The polymicrobial biofilm between the bacterium and fungi was more prominent when *C. albicans* was allowed to form biofilm for 24 h and then *S. epidermidis* was added (Fig. 5h).

### Antibiotic susceptibility of *S. aureus* in monomicrobial (*S. aureus*) and polymicrobial (*S. aureus* plus *C. albicans*) biofilms with planktonic cells *of S. aureus*

**Figure 6:**
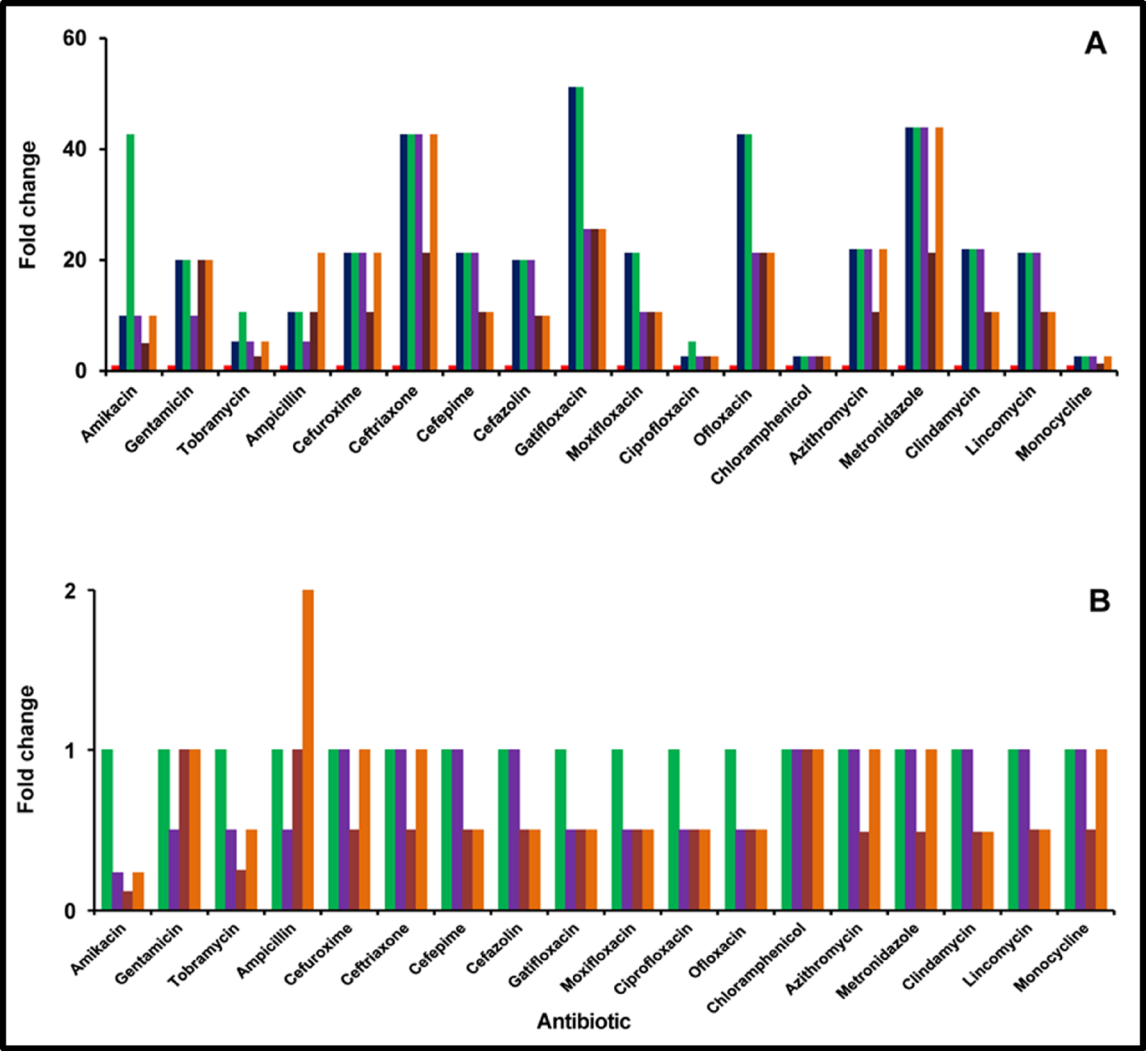
Fold change in minimum biofilm eradication concentration of the antibiotic (MBEC) of monomicrobial (*S. aureus*) and polymicrobial (*S. aureus* plus *C. albicans*) biofilms compared to planktonic cells of *S. aureus* (**A**) and comparison of MBEC between monomicrobial (*S. aureus*) and polymicrobial (*S. aureus* plus *C. albicans*) biofilms (**B**). The effect of the antimicrobial agent was evaluated by the XTT method as described. The coloured bars indicate the following: red square*, S. aureus* in the planktonic phase (24 h); blue square, *S. aureus* in the biofilm phase (24 h); green square, *S. aureus* in the biofilm phase (48 h); purple square, *S. aureus* and *C. albicans* simultaneously incubated to form biofilm (24 h); brown square, *C. albicans* biofilm preformed for 24 h and then *S. aureus* planktonic cells were added; orange square, *S. aureus* biofilm preformed for 24 h and then *C. albicans* planktonic cells were added. Experiments were performed in triplicates.

The concentration at which the XTT method indicated < 0.3 OD_490 nm_ was taken to be the MBEC, since at this concentration none of the cells were viable. The MBEC of *S. aureus* in the biofilm phase was increased several fold (> 2 fold) compared to the planktonic cells for all the 18 different antibiotics that were screened (Supplementary Table 3; Fig. 6A). Amikacin, ceftriaxone, chloramphenicol and ofloxacin at 12 ug/ml were the most effective in the planktonic phase which increased to several fold (32–512 μg/ml) in the biofilm phase at 48 h of biofilm formation (Supplementary Table 3; Fig. 6A). The MBECs of all the 18 antibiotics increased in the polymicrobial biofilm phase irrespective of whether the polymicrobial biofilm was generated by simultaneous incubation or sequential incubation of the bacterium and fungi compared to the planktonic phase *S. aureus* (Supplementary Table 3; Fig. 6 A).

### Antibiotic susceptibility of *S. aureus* in monomicrobial (*S. aureus*) and polymicrobial (*S. aureus* plus *C. albicans*) biofilms

Antibiotic susceptibility of a polymicrobial mixed biofilm of *S. aureus* and *C. albicans* when incubated together for 24 h indicated that the MBEC of 13 antibiotics remained unchanged and in 5 antibiotics the MBEC decreased (Supplementary Table 3) compared to the monomicrobial *S. aureus* biofilm at 24 h. Whereas the MBEC of this biofilm (*S. aureus* and *C. albicans* when incubated together for 24 h) indicated that MBEC of 10 antibiotics remained unchanged and in 8 antibiotics it decreased (Fig. 6B; also see Supplementary Table 3) compared to the monomicrobial *S. aureus* biofilm at 48 h. In a separate experiment when *S. aureus* was added to a preformed 24 h biofilm of *C. albicans* and then tested the MBEC of 4 antibiotics remained unchanged and in 14 antibiotics the MBEC decreased in the mixed polymicrobial biofilm compared to the monomicrobial *S. aureus* biofilm at 24 h (Supplementary Table 3) but MBEC of this biofilm (*S. aureus* was added to a preformed *C. albicans* biofilm) 3 antibiotics remained unchanged and remaining 15 antibiotics showed decreased MBEC compared to the monomicrobial *S. aureus* biofilm at 48 h).In a reverse experiment when *S. aureus* biofilm was preformed for 24 h and then *C. albicans* was added in this mixed polymicrobial biofilm the MBEC of 10 antibiotics remained unchanged, 7 decreased and 1 increased (ampicillin) compared to the MBEC of monobacterial biofilm of *S. aureus* at 24 h (Fig. 6B; also see Supplementary Table 3). But in the 48 h mixed polymicrobial biofilm (*C. albicans* was added to a preformed *S. aureus* biofilm) the MBEC of 7 antibiotics remained unchanged, 10 antibiotics decreased and 1 increased (ampicillin) compared to the monomicrobial *S. aureus* biofilm at 48 h (Fig. 6B; also see Supplementary Table 3).

### Antibiotic susceptibility of *S. epidermidis* in a monomicrobial (*S. epidermidis*) and polymicrobial (*S. epidermidis *plus *C. albicans*) biofilm with planktonic cells of *S. epidermidis*

**Figure 7:**
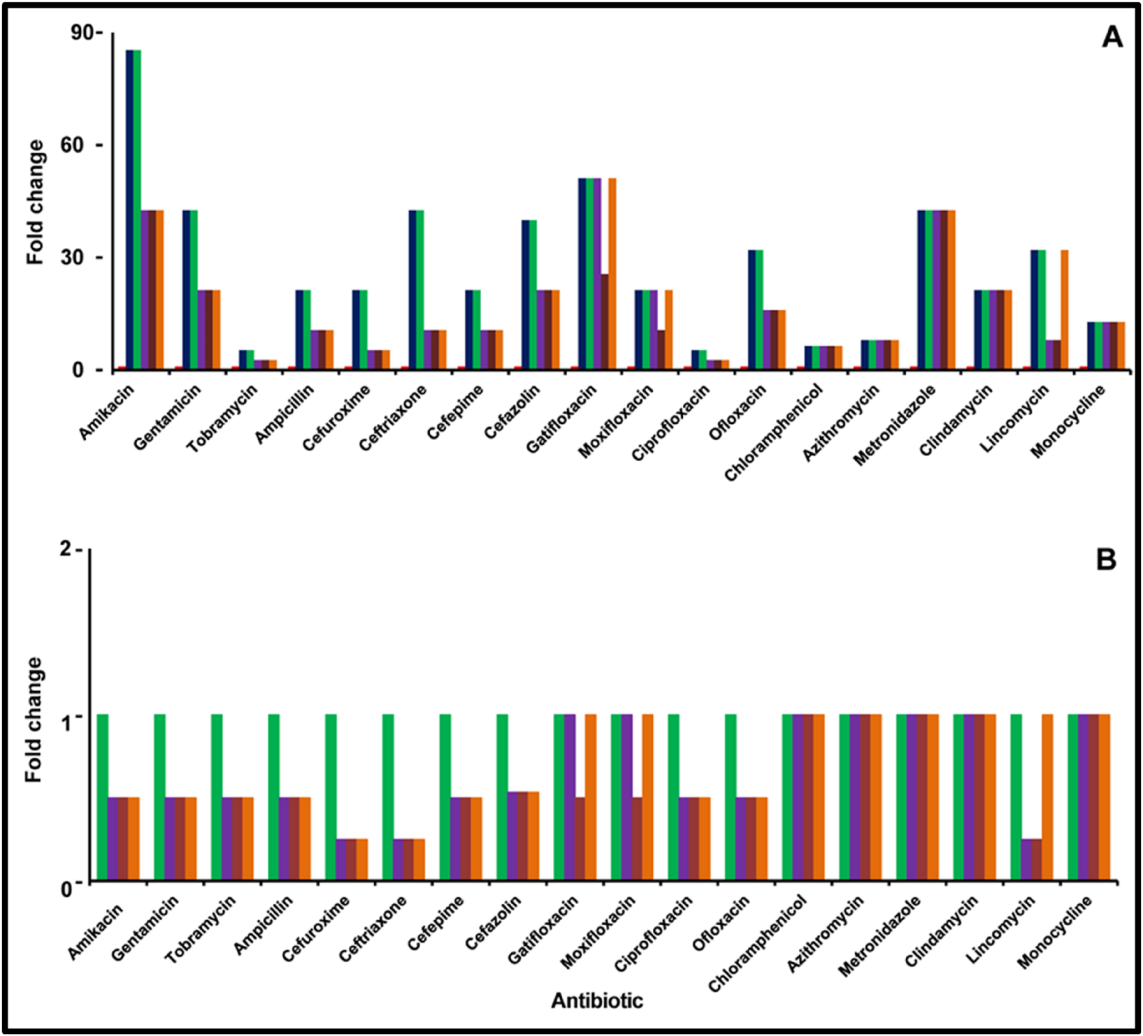
Fold change in minimum biofilm eradication concentration (MBEC) of the antibiotic of monomicrobial (*S. epidermidis*) and polymicrobial (*S. epidermidis* plus *C. albicans*) biofilms compared to planktonic cells of *S. epidermidis* (**A**) and comparison of MBEC between monomicrobial (*S. epidermidis*) and polymicrobial (*S. epidermidis* plus *C. albicans*) biofilms (**B**). The effect of the antimicrobial agent was evaluated by the XTT method as described. The coloured bars indicate the following: red square*, S. epidermidis* in the planktonic phase (24 h); blue square, *S. epidermidis* in the biofilm phase (24 h); green square, *S. epidermidis* in the biofilm phase (48 h); purple square, *S. epidermidis* and *C. albicans* simultaneously incubated to form biofilm (24 h); brown square, *C. albicans* biofilm preformed for 24 h and then *S. epidermidis* planktonic cells were added; orange square, *S. epidermidis* biofilm preformed for 24 h and then *C. albicans* planktonic cells were added. Experiments were performed in triplicates.

*Staphylococcus epidermidis* also showed several fold (> 2-fold) increase in MBEC to the 18 antibiotics in the biofilm phase compared to the planktonic cells (Supplementary Table 4; Fig. 7A) at 24 h and 48 h of biofilm formation. Further*, S. epidermidis* was most susceptible to amikacin, ceftriaxone and cefazolin (MIC: 12 μg/ml) (Supplementary Table 4; Fig. 7A). But when the MBEC of the 18 antibiotics was compared at 48 h of biofilm formation with the biofilm at 24 h, the MBEC at 48 h of biofilm formation remained unchanged for 13 antibiotics and the MBEC of 5 antibiotics increased (Fig. 7; also see Supplementary Table 4).

### Antibiotic susceptibility of *S. epidemidis* in monomicrobial (*S. epidermidis*) and polymicrobial (*S. epidermidis* plus *C. albicans*) biofilms

Further, when the antibiotic susceptibility of a polymicrobial mixed biofilm of *S. epidermidis* and *C. albicans* generated by incubating them together indicated that at 24 h the MBEC of 7 different antibiotics remained unchanged and in 11 antibiotics the MBEC decreased (Supplementary Table 4; Fig. 7B) compared to the monomicrobial *S. epidermidis* biofilm at 24 h and 48 h. In a polymicrobial mixed biofilm in which the bacterium was allowed to form a biofilm for 24 h and then *C. albicans* was added the MBEC of 8 different antibiotics remained unchanged whereas the MBEC of the remaining 10 antibiotics decreased compared to the MBEC recorded for *S. epidermidis* in the biofilm phase at 24 h and 48 h (Supplementary Table 4). In the reverse experiment wherein *C. albicans* formed a biofilm for 24 h and then *S. epidermidis* was added the results indicated that the MBEC to most of the antibiotics (12) decreased whereas MBEC of the remaining 6 antibiotics remained unchanged (chloramphenicol, azithromycin, metronidazole, clindamycin, lincomycin and monocycline) compared to the biofilm of *S. epidermidis* at 24 h and 48 h (Supplementary Table 4).

### Susceptibility of *C. albicans* to antifungal agents in a monomicrobial (*C. albicans*) and polymicrobial (*C. albicans* plus *S. aureus or S. epidermidis*) biofilm

**Figure 8:**
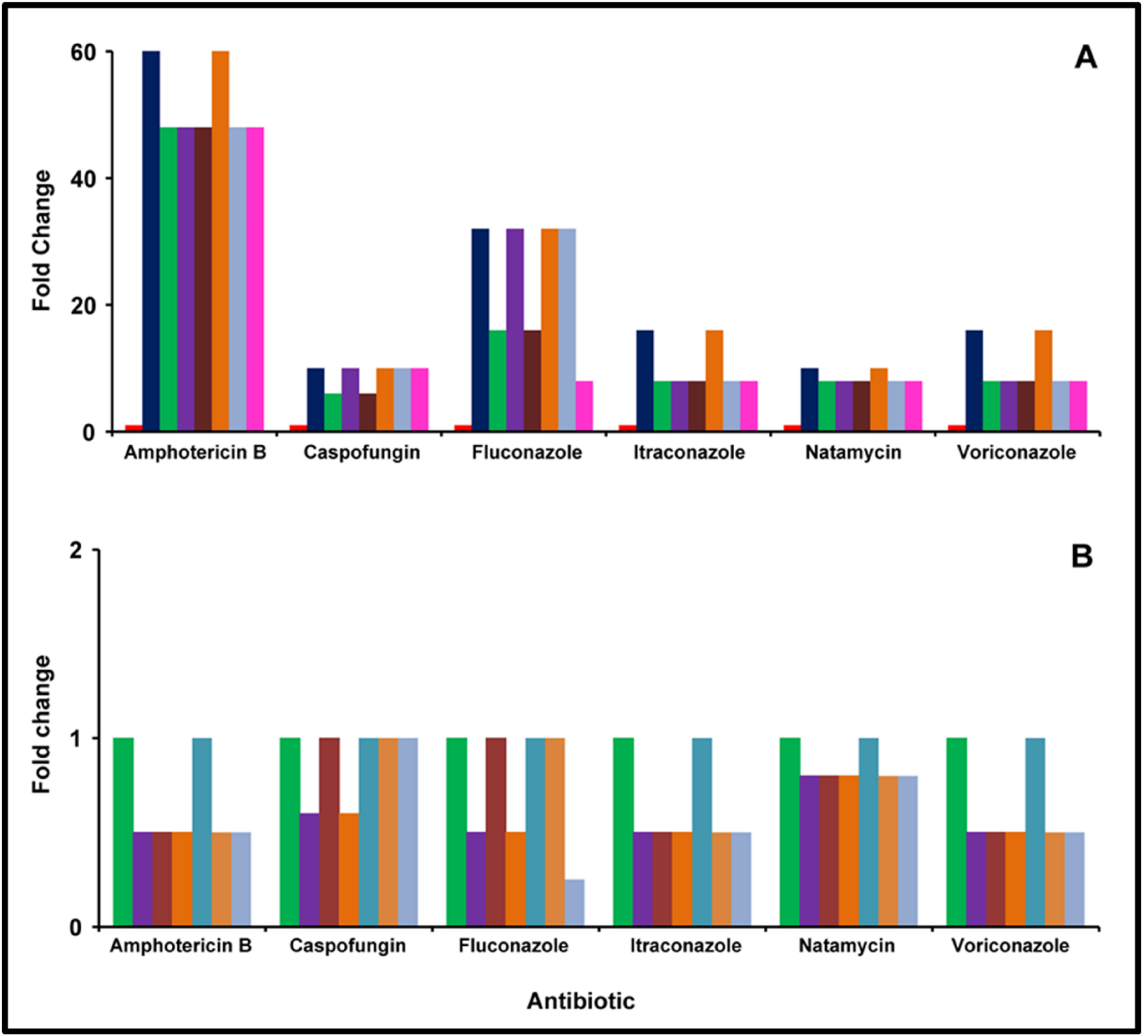
Fold change in the minimum biofilm eradication concentration (MBEC) of the antifungal agent of monomicrobial (*C. albicans*) and polymicrobial (*S. epidermidis* plus *C. albicans* and *S. aureus* plus *C. albicans*) biofilms compared to planktonic cells of *C. albicans* (**A**) and comparison of MBEC between monomicrobial (*C. albicans*) and polymicrobial (*S. epidermidis* plus *C. albicans* and *S. aureus* plus *C. albicans*) biofilms (**B**). The effect of the antimicrobial agent was evaluated by the XTT method as described. The coloured bars indicate the following: red square, *C. albicans* in the planktonic phase (24 h); blue square, *C. albicans* in the biofilm phase (24 h); green square, *S. epidermidis* plus *C. albicans* simultaneously incubated to form biofilm (24 h); purple square, *C. albicans* biofilm preformed for 24 h and then S. epidermidis planktonic cells were added; brown square, *S. epidermidis* biofilm preformed for 24 h and then *C. albicans* planktonic cells were added; orange square, *S. aureus* plus *C. albicans* simultaneously incubated to form biofilm (24 h); light blue square, *C. albicans* biofilm preformed for 24 h and then *S. aureus* planktonic cells were added; pink square, *S. aureus* biofilm preformed for 24 h and then *C. albicans* planktonic cells were added. Experiments were performed in triplicates.

*Candida albicans* in the biofilm phase was less susceptible compared to the planktonic cells to the 6 antifungal agents tested and was most susceptible to caspofungin (MBEC, 20 μg/ml) at 48 h of biofilm growth (Supplementary Table 5). Polymicrobial mixed biofilm of *S. aureus* and *C. albicans* when incubated together (simultaneously) was less susceptible compared to the planktonic cells of *C. albicans* and the MBECs were identical to the MBECs of monomicrobial biofilm of *C. albicans* at 48 h (Fig. 8A, Supplementary Table 5). Further when *C. albicans* biofilm was performed for 24 h and then *S. aureus* cells were added the mixed polymicrobial biofilm after 24 h showed further decrease in MBEC to 4 of the 6 antibiotics whereas susceptibility to caspofungin and fluconazole were similar compared to the mixed simultaneously formed biofilm and monospecies biofilm of *C. albicans* at 48 h (Supplementary Table 5). In reverse experiments when the biofilm was preformed by *S. aureus* and then *C. albicans* was added the MBEC decreased with respect to Fluconazole compared to when *C. albicans* biofilm was preformed (Fig. 8B; Supplementary Table 5) and MBEC of 5 antibiotics decreased (except capsofungin) with respect to simultaneously formed biofilm at 24 h and monospecies biofilm of *C. albicans* at 48 h. Polymicrobial mixed biofilm of *S. epidermidis* and *C. albicans* when incubated together (simultaneously) was less susceptible compared to the planktonic cells of *C. albicans* (Fig. 8A, Supplementary Table 5). MBECs of these simultaneously mixed biofilms exhibited decrease in MBECs for all antibiotics compared to MBECs of monomicrobial biofilm of *C. albicans* at 48 h (Fig. 8B, Supplementary Table 5). In similar experiments, when *C. albicans* biofilm was preformed to which *S. epidermidis* was added the MBECs were identical to that seen when *S. aureus* was added to *C. albicans* biofilm (Fig. 8B, Supplementary Table 5). But when the biofilm of *S. epidermidis* was performed and then *C. albicans* was added the mixed biofilm response was identical to the simultaneously formed biofilm of *C. albicans* and *S. epidermidis* (Fig. 8B, Supplementary Table 5)*.* MBECs for all antibiotics were decreased with respective to monospecies *C. albicans* biofilm at 48 h. But when biofilm of *C. albicans* was performed and *S. epidermidis* was added the MBECs were very similar except for caspofungin and fluconazole which showed increase in MBECs compared to the simultaneously formed biofilm of *C. albicans* and *S. epidermidis* (Fig. 8B, Supplementary Table 5).

## Discussion

The ocular bacteria *Staphylococcus aureus* and *S. epidermidis* and the fungus *C. albicans*^7,32^ have earlier been reported by us to form monomicrobial biofilms ^7,31,32^. Additionally, it is demonstrated for the first time that these ocular bacteria along with the fungus form polymicrobial biofilms. One earlier study had indicated that non-clinical strains of *S. aureus* and *C. albicans* form a polymicrobial biofilm^36^. *C. albicans*, is conducive to polymicrobial biofilm formation with other bacteria such as *Streptococcus mutans*, *Fusobacterium* spp. and *E. coli*^37^. Mixed fungal-bacterial biofilms of *C. albicans* and *E. coli* or *S. aureus* were reported on endotracheal tubes and urinary catheters, and *Aspergillus fumigatus* and *Pseudomonas aeruginosa* in the lungs of cystic fibrosis (CF) patients^38,39^.

Ocular isolates of *S. aureus, S. epidermidis* and *C. albicans* are known to cause several eye diseases. For instance, *S. aureus* causes dacryocystitis, conjunctivitis, keratitis, cellulitis, corneal ulcers, blebitis, and endophthalmitis^40,41^, *S. epidermidis* causes blepharitis and suppurative keratitis and *C. albicans* causes endophthalmitis or choroiditis^42^. In this study, XTT method and the TCP spectrophotometric method consistently demonstrated that *C. albicans* formed polymicrobial mixed biofilms when incubated together (simultaneously) with either *S. aureus or S. epidermidis* (Figs. 1, 2; also see Supplementary Tables 1, 2) or when the bacterium or the fungus were sequentially added to one another after 24 h (Figs. 1, 2; Supplementary Tables 1, 2). The minor discrepancy in the results between the XTT and TCP methods could be attributed to the differences in the two methods. The XTT method reflecting the number of viable and metabolically active cells^43^ whereas in the TCP method crystal violet binds cells as well as matrix components^44^. Several studies have already demonstrated the potential of ocular isolates to form monobacterial biofilms on indwelling ocular medical devices^26,31^ (see “Introduction”). But, we are not aware of any studies on polymicrobial biofilms involving ocular bacteria and fungi as in this study.

Polymicrobial biofilms involving either species of the same genus or species from different kingdoms (such as bacteria and fungi) are probably more dominant in nature^45^. Such polymicrobial biofilms are clinically relevant since they are associated with several ocular infections and infections of the lung, inner ear, urinary tract, oral cavity, wounds, teeth and those that dwell on devices^46,47^. Further, the dual species involved in the formation of polymicrobial biofilms varied depending on the infection^48,49^. The commonly encountered microorganisms in polymicrobial biofilms were *S. aureus*–*Pseudomonas aeruginosa*^50^, *S. aureus–C. albicans*^51^, *S. aureus–C. tropicalis* and *C. tropicalis–S. marcescens*^52^, *Staphylococcus xylosus*–*S. aureus*^53^ etc. It was observed that ocular isolates of *C. albicans, S. aureus* and *S. epidermidis* formed polymicrobial mixed biofilms irrespective of whether the bacteria and fungus were added simultaneously onto the substratum, or the bacterium was added to the preformed fungal biofilm or vice versa. In the latter case, the occurrence of mixed biofilms was indicative that the ocular bacteria and the fungus could penetrate preformed biofilms as reported earlier in mixed biofilms of *S. aureus* and *P. aeruginosa*^50^.

SEM confirmed the formation of monomicrobial and polymicrobial mixed biofilms of ocular *S. aureus, S. epidermidis* and *C. albicans* as judged by clumping of the cells and secretion of EPS (Figs. 4, 5). In the ocular isolates the monomicrobial biofilms at 48 h showed increased clumping of cells and excessive of EPS (Fig. 4a,e, 5a,e) compared to the polymicrobial mixed biofilms (Figs. 4c,d,g,h, 5c,d,g,h) implying that in the dual species the interaction between the taxa may be influencing the biofilm process. Further, when *C. albicans* monomicrobial biofilm was preformed for 24 h and then *S. aureus* was added or vice versa polymicrobial mixed biofilms were denser, hyphae were more prominent, and EPS was clearly visible (Fig. 4d,h).

Confocal microscopy studies indicated that the thickness of the polymicrobial mixed biofilms (*S. aureus* plus *C. albicans* and *S. epidermidis* plus *C. albicans*) increased compared to the monomicrobial biofilms (Fig. 3; also see Supplementary Figs. 1, 2) when the bacteria and fungi were incubated simultaneously to form biofilm. Interestingly it was also observed that when the fungus biofilm was preformed and then either of the bacteria were added to the biofilm the thickness increased. But biofilm thickness did not exhibit significant increase in thickness when the biofilm was preformed by bacteria to which the fungus was added (Fig. 3). The reason for this is not clear but it could imply that the preformed fungal biofilm is not conducive to the establishment of the polymicrobial biofilm by bacteria. Earlier we had shown that theses ocular isolates of *S. aureus, S. epidermidis* and *C. albicans* formed monomicrobial biofilms which increased in thickness with incubation period^7,31^. Several other studies also indicated that biofilm thickness increases with incubation period^54^.

Interactions between the microbes in a polymicrobial biofilm have been implicated in disease progression, in causing an inflammatory state, in inducing collateral damage in the host, in increasing tolerance to biotic stresses due to host and predatory microorganisms and in exhibiting drug resistance and tolerance to antibiotics^10,47,55,56^. Our results confirm that the monomicrobial biofilms exhibited several-fold more resistance to all the antimicrobials tested compared to planktonic cells (Figs. 6, 7, 8; Supplementary Tables 3–5) confirming earlier studies on ocular *S. aureus*^31,41,57^, *S. epidermidis*^31,58^ and *C albicans*^7^. Earlier studies had also indicated that polymicrobial mixed biofilms were more resistant to antimicrobials compared to the monomicrobial biofilms biofilms^36,55,59–61^. For example, a polymicrobial biofilm of *C. albicans* and *S. aureus* was more resistant to vancomycin and daptomycin than as a monoculture^36^. *Staphylococcus epidermidis*, has also been shown to protect *C. albicans* from the action of the antifungal drugs fluconazole and amphotericin B in polymicrobial biofilms^62^. In this study, when the resistance of the polymicrobial mixed biofilms were compared to monomicrobial biofilms the MBEC values either remained unchanged or decreased (Fig. 6, 7, 8; also see Supplementary Table 3) except in one case when *S. aureus* biofilm was performed and then *C. albicans* was added the MBEC of Ampicillin increased (Supplementary Table 3; Fig. 6). Increase in resistance of polymicrobial mixed biofilms to antimicrobials compared to the monomicrobial biofilms has been attributed to poor antibiotic penetration, nutrient limitation, slow growth, stress, formation of persister cells and extracellular biofilm matrix formation^36,62,63^. Interaction between the taxa in a polymicrobial biofilm has also been implicated in enhanced tolerance to antibiotics^64^. For instance in polymicrobial biofilm *C. albicans* enhanced the resistance of *S. aureus*^61,65,66^ to daptomycin and vancomycin^36^. In cystic fibrosis (CF) mixed polymicrobial biofilm with *P. aeruginosa*, and *Inquilinus limosus* or with *Dolosigranulum pigrum* increase the tolerance to most antibiotics^67^. In this study the resistance of the polymicrobial mixed biofilms to several antibiotics was either identical or decreased compared to that of the monomicrobial biofilm. This observation contradicts earlier studies which had also indicated that polymicrobial biofilms are more challenging to treat since they are more resistant to antimicrobial treatment than the corresponding single-species biofilms^21,22^ and corresponding planktonic cells^23^. Identical MBECs to antibiotics in the polymicrobial biofilm would imply that the observed increased thickness of the biofilm in the polymicrobial biofilm (Fig. 3; also see Supplementary Figs. 1, 2) may not be influencing the resistance to antimicrobials. Further, the resistance to antimicrobials decreased in the polymicrobial mixed biofilms compared to the monomicrobial biofilm in many instances (Figs. 6, 7, 8; also see Supplementary Table 3–5) implying that the biotic components (bacteria and fungi) within the biofilm were interacting and making them more sensitive to the drugs^50^. Trizna et al.^50^ had earlier demonstrated that in *S. aureus* and *P. aeruginosa* dual species biofilms a tenfold increase in susceptibility to ciprofloxacin and aminoglycosides (gentamicin or amikacin), was observed compared to monobacterial biofilms. The results imply that strategies used to hack monobacterial biofilms should be equally efficient in targeting microbes in a polymicrobial mixed biofilm.

To the best of our knowledge this is the first study demonstrating that ocular bacteria and fungi possess the potential to form polymicrobial mixed biofilms which exhibit increased resistance to both antibacterial and antifungal agents compared to planktonic cells.

### Relevance of the study

The above results are of relevance to ocular infection treatment. In an ocular clinic handling ocular surface infections the organism that first appears in culture from an ocular sample is the target of treatment. But this may not be the best approach in case of polymicrobial infections since fungal infections are normally detected after a week on culturing, whereas bacterial infections appear within 48 h. Thus, bacteria become the first targets of medication. It is good to start the treatment to target the first detected organism, but one should also look for other organisms which may appear with time, and they also need to be treated. If a polymicrobial infection is not considered or is missed, the outcome may be adversely affected^4^.

## Conclusions

1. Antibiotic and antifungal susceptibility studies confirmed that *S. aureus*, *S. epidermidis* and *C. albicans* in the monomicrobial biofilm phase were several fold more resistant to antimicrobial agents compared to the planktonic phase.
2. Ocular isolates in polymicrobial mixed biofilm phase are also more resistant to antimicrobials compared to the planktonic cells.
3. Ocular isolates in the polymicrobial mixed biofilm phase most often showed no change or decreased resistance to antimicrobials compared to the monomicrobial biofilm phase organisms.
4. Considering that the chosen ocular bacteria and fungus are the etiological agents of several ocular diseases the studies would be very relevant in planning treatment strategies for the eye.

### Limitations

1. The study does not address the cellular-basis of polymicrobial biofilm formation? For instance, when the bacteria or fungi are added to an already formed monomicrobial biofilm how do they attach to the monomicrobial biofilm? Further, it is not clear whether they first attach to the substratum and then to the biofilm or vice versa?
2. Need to study the expression of genes associated with biofilm formation in monomicrobial and polymicrobial biofilms?
3. Need to understand why polymicrobial biofilms exhibit decreased resistance to different antimicrobials compared to the monomicrobial biofilms?
4. Need to extend this study to more combinations of ocular bacteria and fungi.

## Supplementary Information

Supplementary Information.

## Data Availability

All data generated or analyzed during this study are included in this published article and its supplementary information files.
