# Supplementary material for: Polymicrobial biofilms of ocular bacteria and fungi on ex vivo human corneas
Source: Sci Rep. 2022 Jul 8;12:11606. doi: 10.1038/s41598-022-15809-z (PMC9270462; doi:10.1038/s41598-022-15809-z)
Supplement: Supplementary file 1 — Supplementary Information. [file 41598_2022_15809_MOESM1_ESM.docx]

**Supplementary table 1.** Polymicrobial biofilms in ocular *S. aureus, S. epidermidis* and *C. albicans* by the Tissue culture plate method using XTT *

| S. No. | Species/combination | XTT OD _490 nm_ after  24 h of biofilm formation | XTT OD _490 nm_ after  48 h of biofilm formation* |
| --- | --- | --- | --- |
|  | **Monomicrobial biofilm** |  |  |
| 1 | *S. aureus* L1054/2020(2) | 0.45 ± 0.15 | 0.80 ± 0.22 ^a^ |
| 2 | *S. epidermidis* L1058/2020(2) | 0.59 ± 0.10 | 0.98 ± 0.17 ^c^ |
| 3 | *C. albicans* L391/2015 | 0.38 ± 0.06 | 0.86 ± 0.26 ^b^ |
|  | **Polymicrobial biofilm simultaneously formed** |  |  |
| 4 | *S. aureus + C. albicans* | 0.62 ± 0.10 | 1.23 ± 0.10 ^a^ |
| 5 | *S. epidermidis + C. albicans* | 0.96 ± 0.10 | 1.36 ± 0.10 ^b^ |
|  | **Addition to pre-formed 24 h *C. albicans* biofilm** |  |  |
| 6 | *C. albicans + S. aureus* | 0.38 ± 0.06 | 0.72 ± 0.10 |
| 7 | *C. albicans* + *S. epidermidis* | 0.38 ± 0.06 | 0.96 ± 0.15 |
|  | **Addition to pre-formed 24 h bacterial biofilm** |  |  |
| 8 | *S. epidermidis* + *C. albicans* | 0.59 ± 0.10 | 1.685 ± 0.1^c^ |
| 9 | *S. aureus* + *C. albicans* | 0.45 ± 0.15 | 0.98 ± 0.1 |
|  | **Controls** |  |  |
| 10 | *S. aureus* ATCC 25923 (positive control) | 0.65 ± 0.13 | 1.26 ± 0.12 |
| 11 | *E. coli* ATCC 25922 (negative control) | 0.18 ± 0.05 | 0.23 ± 0.12 |

* OD of YPD broth without inoculum (no organism control) was deducted from the OD of monomicrobial/polymicrobial biofilm. Similar superscripts indicate that the values are significantly different (p-value < 0.05) between the indicated rows. Unpaired t-test was used for the calculation of p value. Experiments were performed in triplicates.

**Supplementary table 2.** Polymicrobial biofilms in ocular *S. aureus, S. epidermidis* and *C. albicans* by the tissue culture plate method using crystal violet *

| S. No. | Species/combination | Crystal violet OD_595 nm_ after 24 h of biofilm formation | Crystal violet OD_595 nm_ after 48 h of biofilm formation |
| --- | --- | --- | --- |
|  | **Monomicrobial biofilm** |  |  |
| 1 | *S. aureus* L1054/2020(2) | 0.75 ± 0.13 | 1.28 ± 0.12 ^a^ |
| 2 | *S. epidermidis* L1058/2020(2) | 0.89 ± 0.12 | 1.25 ± 0.14 |
| 3 | *C. albicans L391/2015* | 0.45 ± 0.13 | 0.89 ± 0.12 ^b^ |
|  | **Polymicrobial biofilm simultaneously formed** |  |  |
| 4 | *S. aureus + C. albicans* | 1.35 ± 0.05 | 1.276 ± 0.2 |
| 5 | *S. epidermidis + C. albicans* | 1.54 ± 0.23 | 1.69 ± 0.16 ^b^ |
|  | **Addition to pre-formed 24 h *C. albicans* biofilm** |  |  |
| 6 | *C. albicans + S. aureus* | 0.45 ± 0.13 | 0.82 ± 0.11^a^ |
| 7 | *C. albicans + S. epidermidis* | 0.45 ± 0.13 | 1.2 ± 0.05 |
|  | **Addition to pre-formed 24 h bacterial biofilm** |  |  |
| 8 | *S. aureus + C. albicans* | 0.75 ± 0.13 | 0.9 ± 0.21 |
| 9 | *S. epidermidis* (pre-formed) *+ C. albicans* (Planktonic) | 0.89 ± 0.12 | 1.45 ± 0.11 |
|  | **Controls** |  |  |
| 10 | *E. coli* ATCC 25922 | 0.29 ± 0.10 | 0.32 ± 0.26 |
| 11 | *S. aureus* ATCC 25923 | 1.25 ± 0.12 | 1.8 ± 0.23 |

* OD of YPD broth without inoculum (no organism control) was deducted from the OD of monomicrobial/polymicrobial biofilm. Similar superscripts indicate that the values are significantly different (p-value < 0.05) between the indicated rows. Unpaired t-test was used for the calculation of p value. Experiments were performed in triplicates.

**Supplementary table 3.** Determination of MBEC* of antibiotics for *S. aureus* in a monomicrobial (*S. aureus*) and polymicrobial (*S. aureus* plus *C. albicans*) biofilm using the XTT method (OD _490 nm_)

|  | Antibiotics  (µg/ml) | *S. aureus* planktonic phase  (24 h ) | *S. aureus* biofilm phase  (24 h) | *S. aureus* biofilm phase  (48 h) | *S. aureus* +  *C. albicans* simultaneously incubated to form biofilm (24 h) | *C. albicans*  biofilm pre-formed  for 24 h to which  planktonic  *S. aureus* was  added (24 h) | *S. aureus*  biofilm pre-formed  for 24 h to which planktonic  *C. albicans* was  added (24 h) |
| --- | --- | --- | --- | --- | --- | --- | --- |
| Aminoglycosides | Amikacin | 12 | 120 | 512 | 120 | 60 | 120 |
|  | Gentamicin | 24 | 480 | 480 | 240 | 480 | 480 |
|  | Tobramycin | 24 | 128 | 256 | 128 | 64 | 128 |
| β-lactam | Ampicillin | 24 | 256 | 256 | 128 | 256 | 512 |
| Cephalosporins | Cefuroxime | 24 | 512 | 512 | 512 | 256 | 512 |
|  | Ceftriaxone | 12 | 512 | 512 | 512 | 256 | 512 |
|  | Cefepime | 48 | 1024 | 1024 | 1024 | 512 | 512 |
|  | Cefazolin | 24 | 480 | 480 | 480 | 240 | 240 |
| Fluoroquinolones | Gatifloxacin | 20 | 1024 | >1024 | 512 | 512 | 512 |
|  | Moxifloxacin | 48 | 1024 | >1024 | 512 | 512 | 512 |
|  | Ciprofloxacin | 24 | 64 | 128 | 64 | 64 | 64 |
|  | Ofloxacin | 12 | 512 | 512 | 256 | 256 | 256 |
| Amphenicols | Chloram-phenicol | 12 | 32 | 32 | 32 | 32 | 32 |
| Macrolide | Azithromycin | 48 | >1024 | >1024 | >1024 | 512 | >1024 |
| Nitroimidazole | Metronidazole | 24 | >1024 | >1024 | >1024 | 512 | >1024 |
| Lincosamide | Clindamycin | 48 | >1024 | >1024 | >1024 | 512 | 512 |
|  | Lincomycin | 24 | 512 | 512 | 512 | 256 | 256 |
| Tetracycline | Monocycline | 24 | 64 | 64 | 64 | 32 | 64 |

* MBEC, Minimum biofilm eradication concentration. All experiments were carried out thrice.

**Supplementary table 4.** Determination of MBEC* of antibiotics for *S. epidermidis* in a monomicrobial (*S. epidermidis*) and Polymicrobial (*S. epidermidis* plus *C. albicans*) biofilm using the XTT method (OD _490 nm_)

|  | Antibiotics (µg/ml) | *S. epidermidis* planktonic phase  (24h) | *S. epidermidis* biofilm phase  (24 h) | *S. epidermidis* biofilm phase  (48 h) | *S. epidermidis* plus *C. albicans*  simultaneously incubated to form biofilm  (24 h) | *C. albicans* biofilm pre-formed for 24 h to which planktonic  *S. epidermidis* was added  (24 h) | *S. epidermidis*  biofilm pre-formed  for 24 h to which  planktonic  *C. albicans* was added  (24h) |
| --- | --- | --- | --- | --- | --- | --- | --- |
| Amino-glycosides | Amikacin | 12 | 1024 | >1024 | 512 | 512 | 512 |
|  | Gentamicin | 24 | 1024 | 1024 | 512 | 512 | 512 |
|  | Tobramycin | 48 | 256 | 256 | 128 | 128 | 128 |
| β-lactam | Ampicillin | 48 | 1024 | >1024 | 512 | 512 | 512 |
| Cephalo-sporins | Cefuroxime | 24 | 512 | 512 | 128 | 128 | 128 |
|  | Ceftriaxone | 12 | 512 | 512 | 128 | 128 | 128 |
|  | Cefepime | 48 | 1024 | >1024 | 512 | 512 | 512 |
|  | Cefazolin | 12 | 480 | 480 | 256 | 256 | 256 |
| Fluoroqui-nolones | Gatifloxacin | 20 | 1024 | >1024 | 1024 | 512 | 1024 |
|  | Moxifloxacin | 48 | 1024 | >1024 | 1024 | 512 | 1024 |
|  | Ciprofloxacin | 24 | 128 | 128 | 64 | 64 | 64 |
|  | Ofloxacin | 32 | 1024 | 1024 | 512 | 512 | 512 |
| Amphenicols | Chloram-phenicol | 20 | 128 | 128 | 128 | 128 | 128 |
| Macrolide | Azithro-mycin | 128 | >1024 | >1024 | >1024 | >1024 | >1024 |
| Nitroimidazole | Metronid-azole | 24 | >1024 | >1024 | >1024 | >1024 | >1024 |
| Lincosamide | Clindamycin | 48 | >1024 | >1024 | >1024 | >1024 | >1024 |
|  | Lincomycin | 32 | 1024 | 1024 | 256 | 256 | 1024 |
| Tetracycline | Monocycline | 20 | 256 | 256 | 256 | 256 | 256 |

*MBEC - Minimum Biofilm Eradication concentration (µg/ml). All experiments were carried out thrice.

**Supplementary table 5.** Determination of MBEC* of antifungal agents for *C. albicans* in a monomicrobial (*C. albicans*) and polymicrobial (*S. epidermidis* or *S. aureus* plus *C. albicans*) biofilm

| Antifungal  (µg/ml) | *C. albicans* planktonic phase  (24 h) | *C. albicans* biofilm phase  (48 h) | *S. aureus* plus  *C. albicans* simultaneously incubated to form biofilm  (24 h) | *C. albicans* biofilm pre-formed for 24 h to which planktonic *S. aureus* was added (24 h) | *S. aureus* biofilm pre-formed for 24 h to which planktonic  *C. albicans was added (*24 h*)* | *S. epidermidis* plus  *C. albicans* simultaneously incubated to form a biofilm  (24 h) | *C. albicans* biofilm pre-formed for 24 h to which planktonic  *S. epidermidis* was added  ( 24 h) | *S. epidermidis* biofilm pre-formed for 24 h to which planktonic  *C. albicans* was added  *(*24 h) |
| --- | --- | --- | --- | --- | --- | --- | --- | --- |
| Amphotericin B | 0.5 | 48 | 48 | 24 | 24 | 24 | 24 | 24 |
| Caspofungin | 2 | 20 | 20 | 20 | 20 | 12 | 20 | 12 |
| Fluconazole | 8 | 256 | 256 | 256 | 64 | 128 | 256 | 128 |
| Itraconazole | 8 | 128 | 128 | 64 | 64 | 64 | 64 | 64 |
| Natamycin | 8 | 80 | 80 | 64 | 64 | 64 | 64 | 64 |
| Voriconazole | 4 | 64 | 64 | 32 | 32 | 32 | 32 | 32 |

*MBEC-Minimum Biofilm Eradication concentration (µg/ml). All experiments were carried out thrice.

**
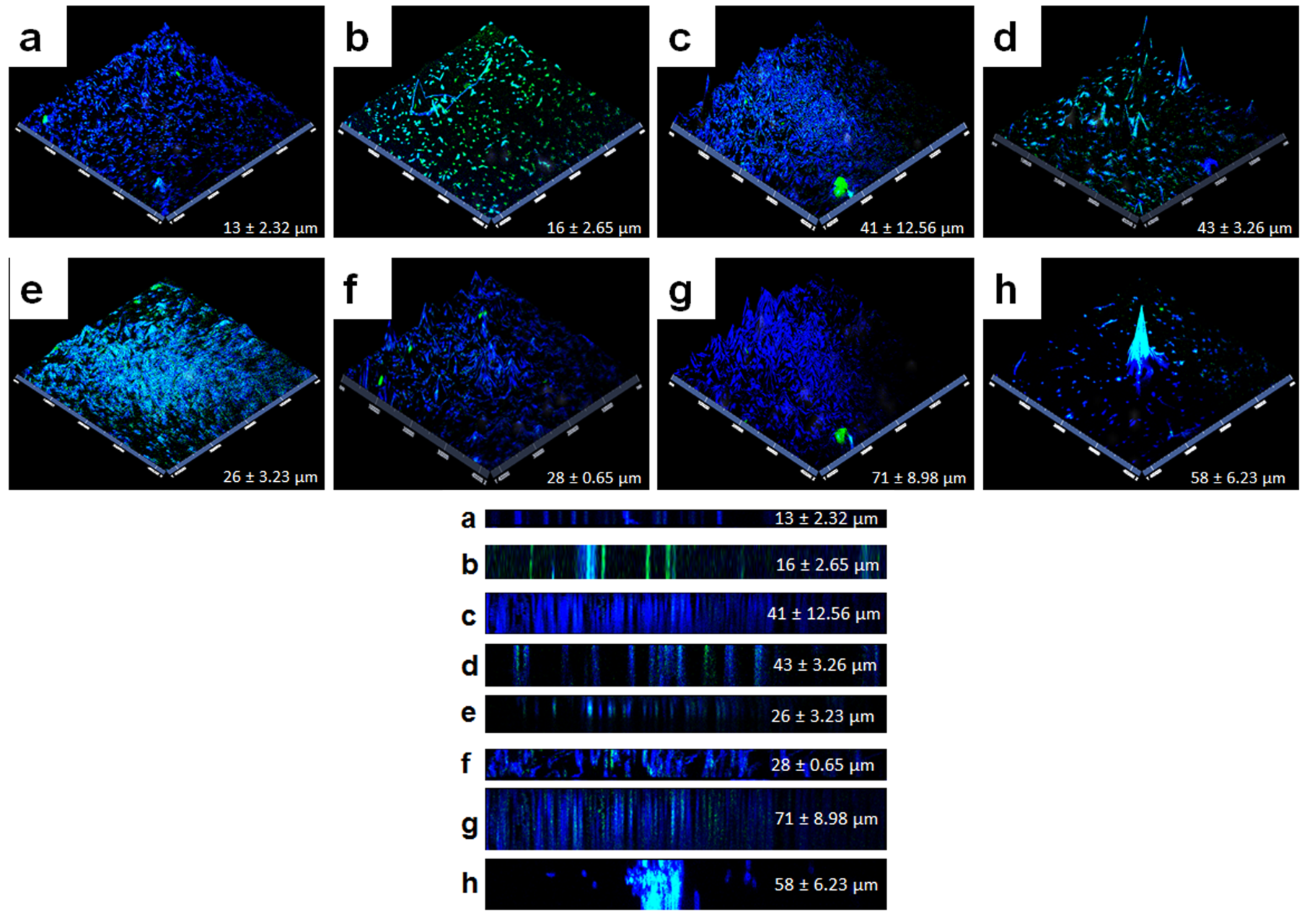
**

**Supplementary figure 1.** Biofilm thickness in monomicrobial and polymicrobial biofilms on human cadaveric corneas using Confocal laser scanning microscope. *S. aureus* biofilm at 24 h (a), *C. albicans* biofilm at 24 h (b), polymicrobial mixed biofilm of *S. aureus* and *C. albicans* grown simultaneously for 24 h (c), preformed biofilm of *S. aureus* for 24 h to which *C. albicans* was added (d), *S. aureus* biofilm at 48 h (e), *C. albicans* biofilm at 48 h (f), polymicrobial mixed biofilm of *S. aureus* and *C. albicans* grown simultaneously for 48 h (g) and preformed biofilm of *C. albicans* for 24 h to which *S. aureus* was added (h). Z stack at the bottom represents the thickness of the biofilm as in a to h. All images were acquired at a magnification of X 20 with zoom scale 2. Experiments were performed in triplicates. The biofilm was stained with Syto9 and Calcofluor white M2R. Viable cells appear green in color and EPS appears blue in color

**
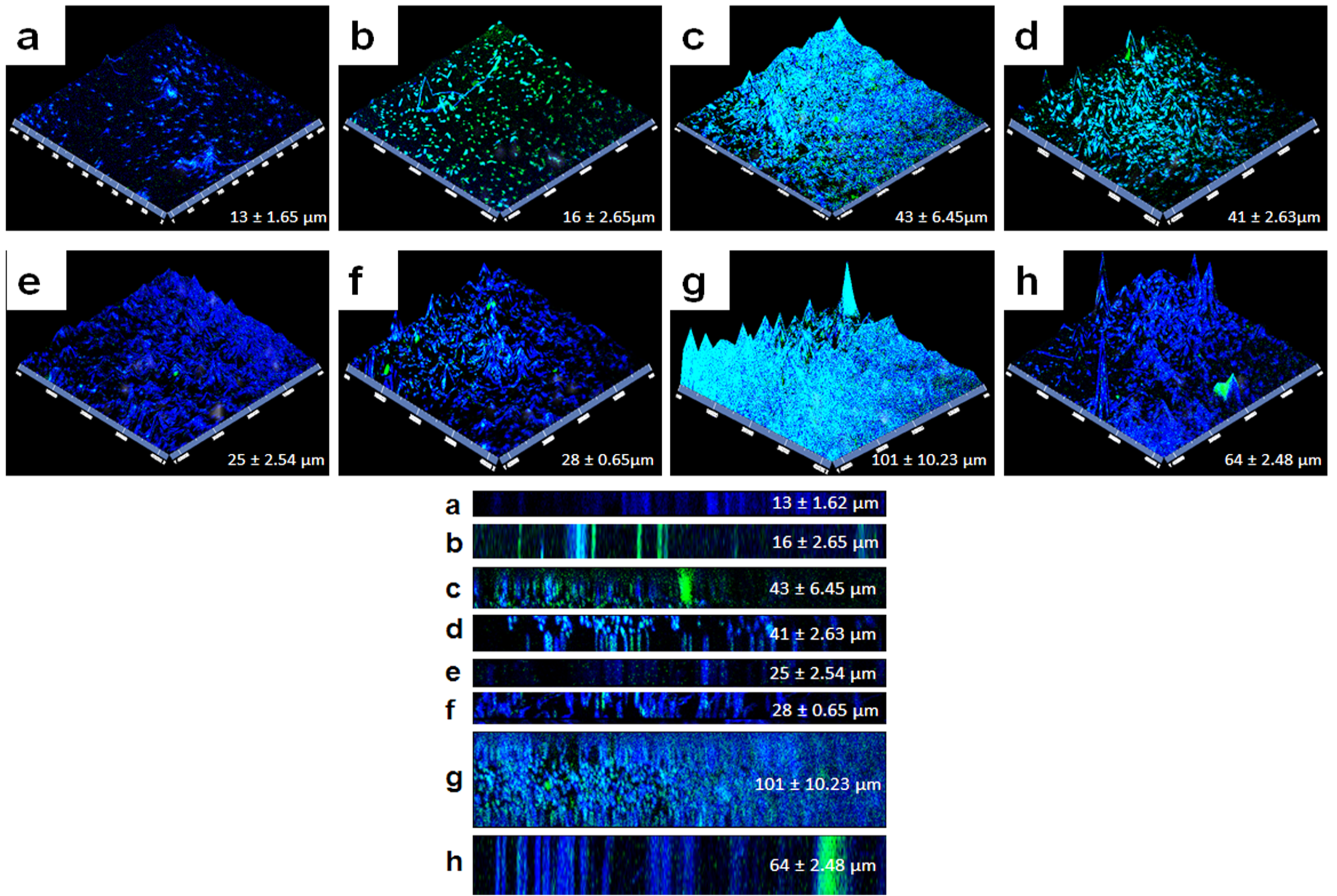
**

**Supplementary figure 2.** Biofilm thickness in monomicrobial and polymicrobial biofilms on human cadaveric corneas using Confocal laser scanning microscope. *S. epidermidis* biofilm at 24 h (a), *C. albicans* biofilm at 24 h (b), polymicrobial mixed biofilm of *S. epidermidis* and *C. albicans* grown simultaneously for 24 h (c), preformed biofilm of *S. epidermidis* for 24 h to which *C. albicans* was added (d), *S. epidermidis* biofilm at 48 h (e), *C. albicans* biofilm at 48 h (f), polymicrobial mixed biofilm of *S. epidermidis* and *C. albicans* grown simultaneously for 48 h (g) and preformed biofilm of *C. albicans* for 24 h to which *S. epidermidis* was added (h). Z stack at the bottom represents the thickness of the biofilm as in a to h. All images were acquired at a magnification of X 20 with zoom scale 2. Experiments were performed in triplicates. The biofilm was stained with Syto9 and Calcofluor white M2R. Viable cells appear green in color and EPS appears blue in color.
